# Coronary sinus unroofing associated with congenitally corrected transposition of the great arteries: a case report

**DOI:** 10.1093/ehjcr/ytaf033

**Published:** 2025-01-28

**Authors:** Federico García-Rodeja Arias, Bernardo López Abel, María Álvarez Barredo, Ana García Campos, José Ramón González Juanatey

**Affiliations:** Cardiology Deparment, Hospital Universitario Santiago de Compostela, Rúa da Choupana s/n, Santiago de Compostela, La Coruña, 15701 Galicia, Spain; Centro de Investigación Biomédica en Red de Enfermedades Cardiovasculares (CIBERCV), Spain; Cardiology Deparment, Hospital Universitario Santiago de Compostela, Rúa da Choupana s/n, Santiago de Compostela, La Coruña, 15701 Galicia, Spain; Centro de Investigación Biomédica en Red de Enfermedades Cardiovasculares (CIBERCV), Spain; Cardiology Deparment, Hospital Universitario Santiago de Compostela, Rúa da Choupana s/n, Santiago de Compostela, La Coruña, 15701 Galicia, Spain; Centro de Investigación Biomédica en Red de Enfermedades Cardiovasculares (CIBERCV), Spain; Cardiology Deparment, Hospital Universitario Santiago de Compostela, Rúa da Choupana s/n, Santiago de Compostela, La Coruña, 15701 Galicia, Spain; Centro de Investigación Biomédica en Red de Enfermedades Cardiovasculares (CIBERCV), Spain; Cardiology Deparment, Hospital Universitario Santiago de Compostela, Rúa da Choupana s/n, Santiago de Compostela, La Coruña, 15701 Galicia, Spain; Centro de Investigación Biomédica en Red de Enfermedades Cardiovasculares (CIBERCV), Spain

**Keywords:** Congenitally corrected transposition, Coronary sinus unroofing, Advanced cardiac imaging, Ventricular septal defect, Case report

## Abstract

**Background:**

Congenitally corrected transposition of the great arteries (ccTGA) is a rare congenital heart defect that is frequently associated with ventricular septal defects (VSDs) and valvular abnormalities. Advanced cardiac imaging techniques are often needed for early detection and detailed study of potential complications during long-term follow-up in these patients.

**Case summary:**

A 20-year-old male, asymptomatic during regular follow-ups, with ccTGA, restrictive subpulmonary VSD, and mild pulmonary stenosis. Latest outpatient evaluations showed progressive biventricular dilation, particularly in the subpulmonary left ventricle. Advanced cardiac imaging, including magnetic resonance imaging (MRI), identified an imbalance in flow between pulmonary and systemic circulations, indicated by a Qp/Qs ratio of 2.6, which could not be attributed to the known restrictive VSD. Additionally, 4D-flow MRI sequences detected low-velocity interatrial flow in the lower atrial segment. Subsequent targeted cardiac computed tomography (CT) confirmed extensive unroofing of the coronary sinus, revealing an unrecognized shunt contributing to the patient’s haemodynamic imbalance.

**Discussion:**

Coronary sinus unroofing is exceedingly rare and often clinically silent, complicating diagnosis. Advanced cardiac imaging (MRI, CT) plays a pivotal role in detecting such anomalies. This case underscores the challenges of diagnosing subtle shunts in complex congenital heart disease and highlights the importance of comprehensive imaging for timely intervention and management.

**Learning points**

- Congenitally corrected transposition of the great arteries often presents with complex structural heart anomalies requiring advanced cardiac imaging for accurate diagnosis and management, as demonstrated in this case of coronary sinus unroofing.
- Despite the patient being largely asymptomatic, the detection of progressive ventricular dilatation and an unexplained high pulmonary/systemic flow ratio underscores the necessity of thorough and continuous monitoring in congenital heart disease to identify rare conditions like coronary sinus unroofing.

## Introduction

Congenitally corrected transposition of the great arteries (ccTGA) is a rare cardiac malformation, occurring in 0.05% of patients with congenital heart disease (CHD).^1^ It is characterized by discordant atrioventricular and ventriculoarterial connections and is often accompanied by other cardiovascular malformations. Common associated defects include ventricular septal defects (VSDs) (68%), valvular and subvalvular pulmonary stenosis (47%), and left atrioventricular (AV) valve regurgitation (57%), with Ebstein-like valve alterations in 32% of patients.^1,2^ Given the structural complexity of these patients, advanced cardiac imaging tests are often required for follow-up.^3,4^ Coronary sinus (CS) variants are not uncommon and can impact ventricular lead implantation for cardiac resynchronization.^5^ However, no previous cases of CS roof defects causing an atrial shunt have been described. This may be the first reported case of a CS unroofing associated with ccTGA.

## Summary figure

Multimodality imaging using advanced imaging techniques for the diagnosis of coronary sinus unroofing. (*A*) 4D-flow sequence of cardiac magnetic resonance imaging showing blood flow rotating within the left atrium and entering the coronary sinus and finally draining into the right atrium. (*B* and *C*) Contrast-enhanced CT images, sequential slices of the short axis of the coronary sinus. Dilated coronary sinus with intact roof separating it from the left atrial cavity in *B*. Coronary sinus without roof in *C*.

**Figure:**
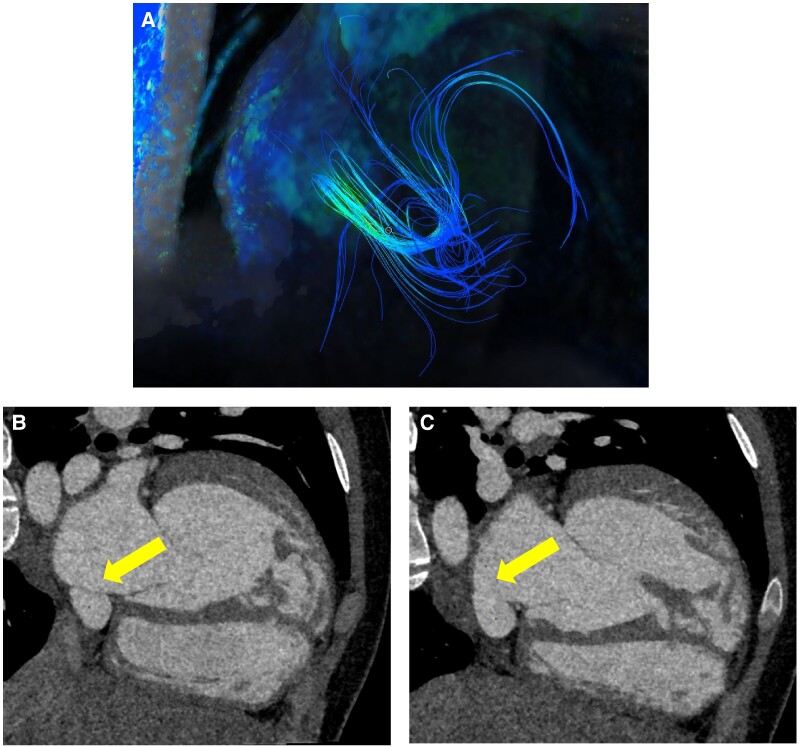


## Case summary

This is a 20-year-old Caucasian male on regular outpatient follow-up for ccTGA associated with a restrictive subpulmonary VSD and mild pulmonary valvular stenosis. Since neonatal diagnosis, he has been monitored every six months, remaining mostly asymptomatic. Notable events during childhood include two hospital admissions for pneumonia, with favourable recovery, and sporadic asthmatic exacerbations. He has no other medical comorbidities and no family history of relevant diseases. Despite these incidents, he has maintained an active lifestyle without sports limitations.

During paediatric and early adulthood follow-up, multiple 24-h Holter monitor recordings detected only isolated supraventricular and ventricular extrasystoles. His condition was monitored through serial transthoracic echocardiograms (TTE) and magnetic resonance imaging (MRI) scans, which remained stable until the last two follow-up visits over the last year. At these visits, the patient exhibited no signs of heart failure, maintained an oxygen saturation above 97%, and had a known pansystolic murmur in the pulmonary area, graded III/VI. Blood tests showed a haemoglobin level of 16.5 g/dL, normal renal function, and an N-terminal pro–B-type natriuretic peptide (NT-proBNP) level of 80.0 pg/mL. However, TTE revealed progressive ventricular dilation, more pronounced in the subpulmonary left ventricle (LV), which had previously maintained normal dimensions, while biventricular function remained preserved.

**Figure 1:**
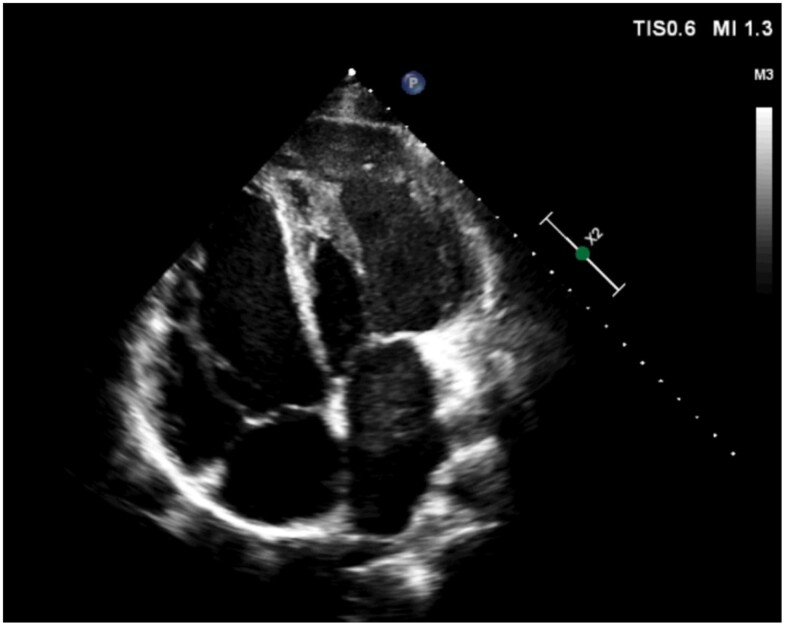
Transthoracic echocardiogram image. Four-chamber view in end-diastole showing biventricular dilatation with displacement of the interventricular septum towards the morphologically right ventricle in systemic position.

**Figure 2:**
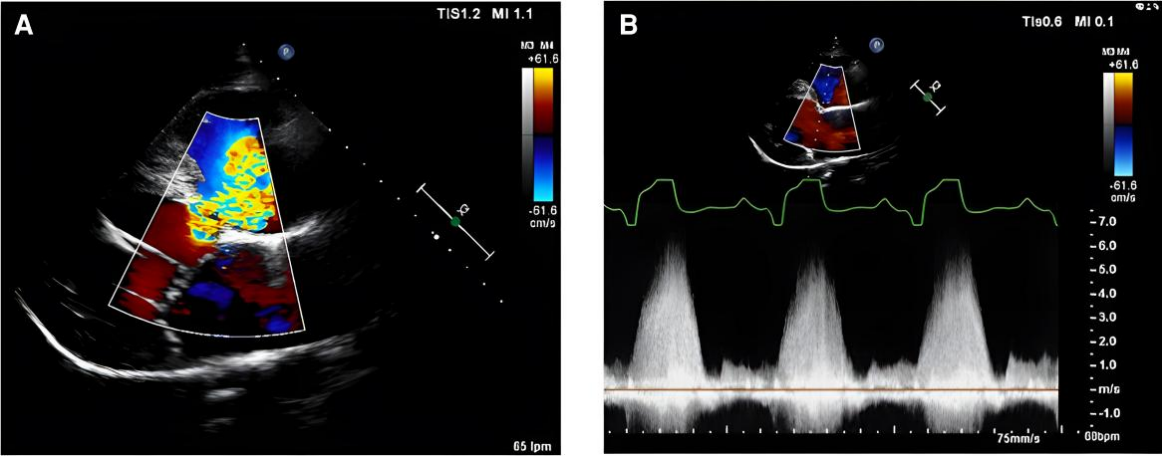
Transthoracic echocardiogram image of subpulmonary ventricular septal defect (VSD). (*A*) Parasternal long-axis plane with colour Doppler showing interventricular shunt with unidirectional flow from the subaortic right ventricle to the subpulmonary left ventricle. (*B*) Continuous Doppler flow of the VSD with a maximum velocity of 6 m/s.

The most recent TTE showed a dilated systemic right ventricle (RV) with preserved global systolic function, an end-diastolic volume of 177 mL (152 mL two years earlier), an end-systolic volume of 74 mL, and an ejection fraction (EF) of 58% by the Simpson method, with mild left AV valve regurgitation. The subpulmonary LV was dilated, with an end-diastolic diameter of 60 mm (previously 51 mm) and a midventricular diameter of 43 mm, maintaining a preserved shortening fraction (mitral annular plane systolic excursion of 33 mm) (*Figure 1* and Supplementary material). No evidence of right AV valve regurgitation was found. The known subpulmonary VSD had a peak velocity of 6 m/s and a maximum gradient of 144 mmHg (*Figure 2*).

A cardiopulmonary exercise test was performed, showing excellent subjective exertion capacity and normal cardiopulmonary behaviour, exceeding age-related expectations. The patient reached a maximum load of 224 W (2.7 W/kg, 129% of the theoretical expected value), with a maximum oxygen consumption of 36.3 mL/min/kg. A cardiac MRI scan performed for volume and flow assessment showed subpulmonary LV dilatation, with an indexed ventricular volume of 131 mL/m², while maintaining preserved systolic function (EF of 64%). The systemic RV also showed dilatation, with an indexed ventricular volume of 161 mL/m² (109 mL/m² in the cardiac MRI scan performed five years earlier) and preserved systolic function (EF of 58%). Direct flow analysis highlighted a 135 mL difference in effective stroke volume between the pulmonary and aortic valves, with a pulmonary/systemic flow ratio (Qp/Qs) of 2.6. Regarding the subpulmonary VSD, predominantly systolic left-to-right flow was observed, with a diameter of 10 × 7 mm and a flow calculated directly by the 4D-flow sequence of 2.5 L/min or 34 mL/beat, with a peak velocity of 4.5 m/s. A left-to-right shunt was identified in the lower region of the interatrial septum in both 4D-flow and cine axial sequences (*Summary figure* and Supplementary material). However, anatomical MRI sequences were inconclusive due to respiratory artefacts. The estimated shunt output exceeded 1.5 L/min, with a peak velocity of 1.3 m/s.

**Figure 3:**
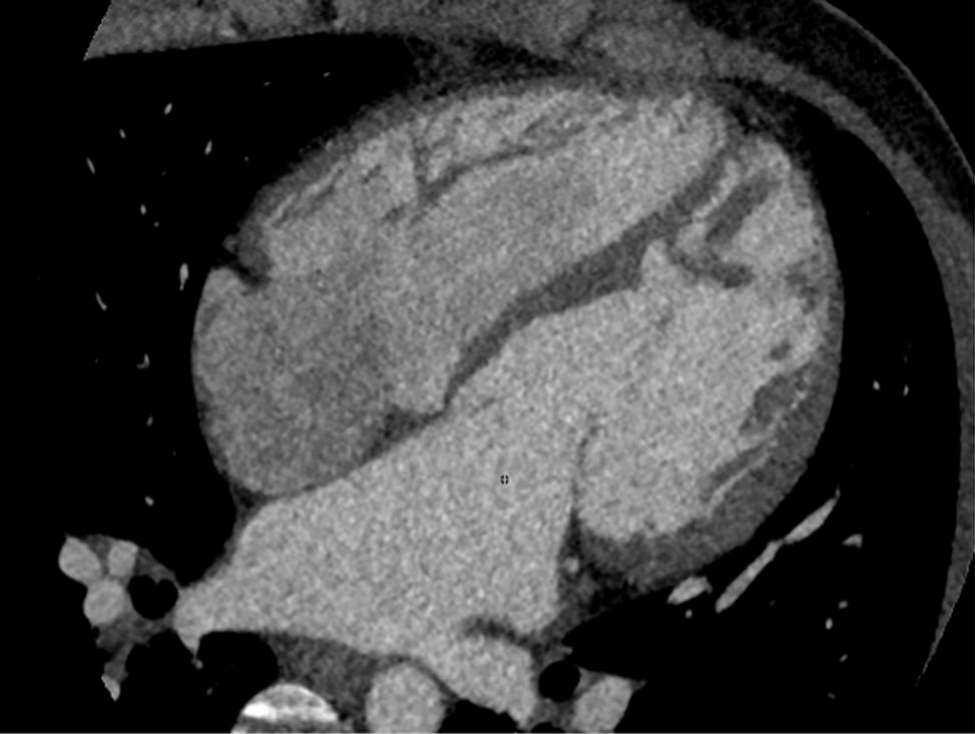
Contrast-enhanced CT image, apical four-chamber plane showing atrioventricular discordance and dilatation of both ventricular cavities.

Given the excessive magnitude of the shunt relative to the VSD dimensions and its restrictive behaviour, a cardiac computed tomography (CT) scan (*Figure 3*) was performed to rule out anomalous venous drainage and evaluate a probable atrial septal defect suspected from the analysis of 4D-flow MRI sequences. The study confirmed normal pulmonary venous drainage through four veins leading to the left atrium and an intact atrial septum. However, axial CS sections (*Summary figure*) showed CS dilation and extensive unroofing at the left atrial portion, confirming the diagnosis. This anomaly was responsible for the haemodynamic imbalance between the pulmonary and systemic circulations, leading to progressive cardiac chamber dilation.

The patient was informed of the diagnosis and potential long-term complications. Consequently, based on the available information and the current recommendations of the European Society of Cardiology guidelines for the management of adult CHD,^6^ and with informed consent, he is being considered for combined surgical closure of the wide CS unroofing and subpulmonary VSD, following the invasive exclusion of pulmonary arterial hypertension.

## Discussion

Coronary sinus unroofing is an extremely rare cardiac anomaly, accounting for <1% of atrial septal shunts. Its diagnosis is challenging due to its low prevalence and variable, often non-specific clinical presentation, with subtle or absent symptoms, as in this case.

Physical examination findings may be inconclusive and go unnoticed in routine screening tests, even with cardiac cavity dilatation, if there is no prior suspicion.^7^ Therefore, advanced cardiac imaging tests, such as CT and MRI,^8^ including 4D-flow sequences,^9^ are essential for establishing suspicion and confirming the diagnosis using specific acquisition and post-processing techniques. Surgical repair has traditionally been the primary treatment due to its anatomical complexity.^10^ However, in recent years, percutaneous closure techniques using Amplatzer devices or covered stents have been explored.^11,12^ Nonetheless, no evidence supports their use in CHD cases like this one.

The complexity of this case lies not only in its rarity, since an unroofed CS is a rare congenital defect, but also in the unique challenges posed by its coexistence with ccTGA. Furthermore, the patient remained asymptomatic despite progressive subpulmonary LV dilatation and a known VSD, which alone did not explain such a high Qp/Qs flow ratio. This raised suspicion of another shunt. Magnetic resonance imaging cine sequences and CT imaging ruled out anomalous systemic and pulmonary venous drainage and a persistent left superior vena cava as causes of CS dilatation. Finally, 4D-flow MRI confirmed a left atrium-to-CS and right atrium shunt, leading to the diagnosis of extensive CS unroofing, later confirmed by cardiac angioCT.

Accurate CHD diagnosis requires specialized personnel and a multimodal approach using advanced imaging such as cardiac MRI and angioCT.^3,4^ Continuous surveillance is crucial for early detection of subtle changes in ventricular morphology, function, or valves.

In summary, CS unroofing is a rare anomaly that may be missed by conventional follow-up, highlighting the importance of clinical suspicion in unexplained haemodynamic imbalances.

## Supplementary Material

ytaf033_Supplementary_Data

## Data Availability

The data underlying this article will be shared on reasonable request to the corresponding author.

## Lead author biography

Federico García-Rodeja is currently a cardiology resident at Santiago de Compostela University Hospital in Spain. He completed his medical school training at the Santiago de Compostela University in 2018. His scientific interest is in electrophysiology.

## Notes

**Consent:** The authors confirm that consent for submission and publication of this case, including images and associated text, has been obtained from the patient in line with COPE guidance.

**Funding:** None declared.
